# Association between sleep quality and cognitive impairment in older adults hypertensive patients in China: a case–control study

**DOI:** 10.3389/fpubh.2024.1446781

**Published:** 2024-11-01

**Authors:** Shunxin Lv, Huachen Jiao, Xia Zhong, Ying Qu, Mengdi Zhang, Rui Wang, Donghai Liu

**Affiliations:** ^1^The First Clinical Medical School, Shandong University of Traditional Chinese Medicine, Jinan, Shandong, China; ^2^Department of Cardiology, Affiliated Hospital of Shandong University of Traditional Chinese Medicine, Jinan, Shandong, China; ^3^Institute of Child and Adolescent Health, School of Public Health, Peking University, Beijing, China; ^4^School of Laboratory Animal & Shandong Laboratory Animal Center, Shandong First Medical University & Shandong Academy of Medical Sciences, Jinan, Shandong, China

**Keywords:** older adults hypertension, sleep quality, cognitive impairment, physical activity, mediation analysis

## Abstract

**Background:**

Previous studies have found that poor sleep quality promotes the occurrence of cognitive impairment (CI), but this relationship has been rarely reported in older adults hypertensive patients. The purpose of this study was to investigate the relationship between sleep quality and CI in older adults hypertensive patients and the mediating effect of sleep quality between physical activity (PA) and CI.

**Methods:**

A total of 2072 older adults hypertensive patients were included in this case–control study. Five hundred and eighteen older adults hypertensive patients with CI were matched 1:3 by age and sex to 1,554 older adults hypertensive patients with normal cognitive function. The International Physical Activity Questionnaire-Long Form, Pittsburgh Sleep Quality Index, and Mini-Mental State Examination were used to evaluate PA intensity, sleep quality, and cognitive function in older adults hypertensive patients. Multivariate logistic regression and the mediation package in R Language were used to analyze the relationship between sleep quality and CI and the mediating effect of sleep quality between PA intensity and CI in older adults hypertensive patients.

**Results:**

After adjusting for all confounding factors, sleep quality was positively correlated with CI in older adults hypertensive patients (OR = 2.565, 95%CI: 1.958–3.360, *p* < 0.001), and this relationship also existed in the older adults hypertensive patients with education levels of primary school and below and junior high school and above (OR = 2.468, 95%CI: 1.754–3.473, *p* < 0.001; OR = 2.385, 95%CI: 1.367–4.161, *p* = 0.002). In addition, sleep quality mediated part of the mediating effect between PA intensity and CI in older adults hypertensive patients (Za*Zb: - 17.19339; 95%CI: −0.37312, −0.04194).

**Conclusion:**

Poor sleep quality was associated with the occurrence of CI in older adults hypertensive patients, and this relationship also existed in older adults hypertensive patients with education levels of primary school and below and junior high school and above.

## 1 Introduction

High systolic blood pressure has become a major risk factor for the global burden of disease by 2017 (1). By 2019, approximately 49% of men and 59% of women globally have been hypertensive patients (2). Hypertension is not only associated with an increased risk of cardiovascular disease, chronic kidney disease, stroke, and other diseases (3), but also increases the risk of cognitive impairment (CI) (4). Studies have found that the prevalence of hypertension with mild cognitive impairment (MCI) is 30% (5), and the prevalence of CI in hypertensive patients in China is 37.6% (6). CI has aggravated the economic burden of patients and increased the rehospitalization rate of patients (7, 8), which has become a public health problem plaguing the world. Unfortunately, there is no specific drug for the treatment of CI (9). Therefore, it is very important to prevent and delay the occurrence of CI, especially for hypertensive patients.

Sleep plays a variety of basic biological functions in the human body, such as the organism’s maintenance, repair, and construction (10). Studies have found that sleep has many benefits for human health. It is beneficial to the formation and consolidation of human brain memory (11), the regulation of emotion (12), the balance of energy metabolism (13), the synthesis of biological macromolecules (14), and the clearance of metabolites (15). Poor sleep quality is an important symptom of sleep disorders and reflects the nature and severity of sleep deprivation (16, 17). Recent studies have found that poor sleep quality can promote cognitive decline and there was also a synergistic effect between poor sleep quality and hypertension. Two meta-analyses found that poor sleep quality was associated with MCI (18); the sleep quality of older adults patients with MCI was lower than that of healthy older adults people (19). In addition, a meta-analysis study and a cross-sectional study found that poor sleep quality was associated with hypertension, and hypertension promoted poor sleep quality (20, 21). However, there are few studies on the association between sleep quality and CI in older adults hypertensive patients. Physical activity (PA) is closely related to human health. Studies have found that PA is associated with improvements in sleep quality and cognitive function (22–24), but the relationship among them is rarely reported.

This study aimed to analyze the relationship between sleep quality and CI in older adults hypertensive patients, and whether sleep quality mediates the association between PA and CI.

## 2 Materials and methods

### 2.1 Study design and population

#### 2.1.1 Source of study cases

The data of this case–control study came from the hypertension electronic community follow-up system of the Affiliated Hospital of Shandong University of Traditional Chinese Medicine. The system collected hypertension patients from outpatients or wards of 9 hospitals which were the Affiliated Hospital of Shandong University of Traditional Chinese Medicine, Jinan Hospital of Traditional Chinese Medicine, Jinan Minzu Hospital, Qingzhou People’s Hospital, Penglai Hospital of Traditional Chinese Medicine, Tai ‘an First People’s Hospital, Guangrao County Hospital of Traditional Chinese Medicine, Qufu Hospital of Traditional Chinese Medicine, and Weifang Hospital of Traditional Chinese Medicine. The matched case–control sample size was calculated using the following formula:


$$M=\frac{{\left(\frac{u_{\alpha }}{2}+u_{\beta }\sqrt{\frac{\mathrm{OR}}{1+\mathrm{OR}}\left(1-\frac{\mathrm{OR}}{1+\mathrm{OR}}\right)}\right)}^{2}}{{\left(\frac{\mathrm{OR}}{1+\mathrm{OR}}-\frac{1}{2}\right)}^{2}\left(p_{0}q_{1}+p_{1}q_{0}\right)}$$

Among them, u_α_ was 1.96, u_β_ was 1.28, OR was 2.16, p_0_ was 0.15, p_1_ was 0.28, q_0_ was 0.85, and q_1_ was 0.72 (25, 26). Since 1:3 matching was performed in this study, it was calculated that the case group was 269 (1.2 × M) at least required, and the control group was 807 at least required. In this study, the hypertensive cases of the system from May 2022 to February 2024 were exported and summarized into an Excel table, with a total of 5,118 cases. After excluding 1714 hypertensive cases with severe data loss and age < 60 years, the total number of included cases was 3,404. The diagnostic criteria for hypertension meet one of the following criteria: (1) office systolic blood pressure (SBP) ≥ 140 mmHg and/or diastolic blood pressure (DBP) ≥ 90 mmHg on 3 times on different days without using antihypertensive drugs.; (2) SBP ≥ 135 mmHg and/or DBP ≥ 85 mmHg measured at home 3 times on different days without using antihypertensive drugs; (3) 24 h ambulatory average blood pressure ≥ 130/80 mmHg; daytime ≥135/85 mmHg; nighttime ≥120/70 mmHg. (4) The patient has a history of hypertension and is currently taking antihypertensive drugs. Although the blood pressure is <140/90 mmHg, it should still be diagnosed as hypertension (27). Cognitive function was assessed by Mini-mental State Examination (MMSE). MMSE score between 27 and 30 was defined as normal cognitive function, and MMSE score less than 27 was defined as CI (28).

#### 2.1.2 Inclusion criteria

The inclusion criteria were as follows: (1) age ≥ 60 years old; (2) patients with essential hypertension; (3) signing informed consent; (4) voluntarily completing the collection of hypertension-related information.

#### 2.1.3 Exclusion criteria

The exclusion criteria were as follows: (1) patients with various types of secondary hypertension; (2) vascular dementia or cognitive decline due to vascular factors; (3) new drug clinical trials in the past 3 months; (4) pregnant women, pre-pregnant women, and lactating women; (5) comorbid psychosis and/or psychotropic substance use disorder or dependence; (6) malignant tumor and liver, kidney, and heart function are seriously damaged.

#### 2.1.4 Matching of cases and study indicators

The older adults hypertensive patients with CI and normal cognitive function were matched 1:3 by age and gender, and a total of 2072 older adults hypertensive patients were enrolled, including 518 hypertensive patients with CI and 1,554 hypertensive patients with normal cognitive function. In this study, the baseline data of older adults hypertensive patients were collected, including basic information: age, gender (male/female), education level (primary school and below/junior high school and above), type of work (physical work mainly, mental work mainly, and both), smoking (no/yes), alcohol drinking (no/yes), course of hypertension, hypertension classification (grade 1, 2, and 3), systolic pressure at ordinary times, diastolic pressure at ordinary times, waist-to-hip ratio (WHR): Waist circumference/hip circumference, body mass index (BMI): weight/height^^2^, hypertension medication (no/yes): calcium channel blocker (CCB), diuretic, angiotensin-converting enzyme inhibitor or angiotensin receptor blocker (ACEI or ARB), beta-blockers, sympathetic nerve inhibitor; auxiliary examination: serum metabolic indicators (fasting blood glucose (FBG), triglyceride (TG), total cholesterol (TC), high density lipoprotein cholesterol (HDL-C), low density lipoprotein cholesterol (LDL-C), serum creatinine (Scr)), left ventricular end-diastolic diameter (LVEDD), right ventricular end-diastolic diameter (RVEDD), left atrial diameter (LAD), and subjective evaluation scale: International Physical Activity Questionnaire-Long Form (IPAQ-L), Pittsburgh Sleep Quality Index (PSQI), MMSE.

#### 2.1.5 Ethics statement

This study followed the principles of the Declaration of Helsinki and was approved by the Ethics Committee of the Affiliated Hospital of Shandong University of Traditional Chinese Medicine ((2023) Review No. (109) -KY). Hypertensive patients in the electronic community follow-up system of hypertension signed written informed consent.

### 2.2 Assessment of cognitive function

The Chinese version of MMSE can effectively and reliably assess the cognitive function of the Chinese population (29). The MMSE contains seven simple task domains of time orientation, place orientation, immediate memory, attention and calculation, delayed memory, language ability, and visuospatial ability and is divided into five sections: orientation (10 points), memory (3 points), attention and numeracy (5 points), recall (3 points), and language (9 points). Scores range from 0 to 30, with higher scores indicating better cognitive function (30).

### 2.3 Assessment of sleep quality

PSQI has good reliability and validity in evaluating sleep quality in the Chinese population. The scale consists of 19 items, including 7 elements: (1) subjective sleep quality; (2) sleep latency; (3) sleep duration; (4) sleep efficiency; (5) sleep disorders; (6) sleeping pills; and (7) daytime dysfunction. Each component is scored on a scale of 0 to 3, and the sum of the scores from the seven components produces a subjective sleep-quality score (which ranges from 0 to 21 points). The PSQI total score > 5 was considered poor sleep quality (31).

### 2.4 Assessment of physical activity

IPAQ-L can effectively and reliably assess PA intensity in the Chinese population (32, 33). The IPAQ-L is used to assess daily work, daily living, daily transportation, sports, and recreational activities in the past 7 days. IPAQ-L data are converted into metabolic equivalent task scores (METs) for each dimension or intensity of PA. PA per week (MET-min/week) is calculated by multiplying the total number of minutes per week of each activity by the specific METs for that activity and then summing the total metabolic equivalent task (MET) for each activity. The METs are 3.3 for walking, 4 for moderate activity, 6 for cycling, and 8 for vigorous activity. PA is classified into three categories: (1) Low intensity (category 1): this is the lowest level of PA. Patients do not meet grade 2 or 3 criteria. (2) Moderate intensity (category 2): one of the following three criteria: ① vigorous activity for at least 20 min per day for more than 3 days; ② do at least 30 min of moderate intensity activity or walking daily for more than 5 days; ③ 5 or more days of any combination of walking, moderate or vigorous intensity activity to achieve a minimum of at least 600 MET-min per week. (3) High intensity (category 3): one of the following two criteria: ① vigorous activity for at least 3 days, accumulating at least 1,500 MET-min per week; ② walking at least 3,000 MET-min per week for 7 consecutive days at moderate or vigorous intensity (34).

### 2.5 Measurement of cardiac structure

By a professional cardiac color Doppler technician in the hospital using the American GE color Doppler ultrasound instrument, LVEDD and RVEDD were measured at the level of the mitral tendon cable in parasternal left ventricular long-axis view at end-diastole and LAD which was also the anteroposterior diameter of the left ventricle was measured by taking a vertical line from the posterior wall of the distal aorta to the posterior wall of the left atrium from the parasternal long axis view of the left ventricle at the end of ventricular systole (35).

### 2.6 Measurement of serum metabolic indexes

Medically trained nurses collected blood samples from patients who had fasted for more than 12 h at each hospital and blood samples of patients were centrifuged until patient serum was obtained. The levels of FBG, TG, TC, HDL-C, and LDL-C were determined by enzymatic and homogeneous methods on an automatic biochemical analyzer (Roche Cobas 8,000) and matching kits.

### 2.7 Statistical analysis

All statistical analyses were performed using SPSS software (version 26.0; SPSS Inc., Chicago, IL, USA), GraphPad Prism software (version 9.0.0; GraphPad Software, San Diego, CA, USA), and R Language software (version 4.3.3; R Language Software, Auckland, NI, NZ). Quantitative data were expressed as mean ± standard deviation or median and interquartile range, and a comparison between groups was performed using the t-test or Mann–Whitney U test. Qualitative data were expressed as percentages, and a comparison between groups was performed using the Chi-square test. Spearman correlation coefficient was used to analyze the correlation between sleep quality and the total score of MMSE components. Multivariate logistic regression was used to analyze the relationship between sleep quality and cognitive function. The Mediation package in R Language was used to analyze the mediating effect of sleep quality between PA intensity and CI in older adults hypertensive patients. Two-sided *p*-values of less than 0.05 were considered to indicate statistical significance.

## 3 Results

### 3.1 Baseline differences between the CI group and the normal cognitive function group in older adults hypertensive patients

Table 1 shows the differences between the CI group and the normal cognitive function group in the basic information, auxiliary examinations, hypertension medication use, and subjective evaluation scales in older adults hypertensive patients. The results showed that compared with the normal cognitive function group, the CI group had a higher proportion of education level of primary school and below, work type with physical work mainly, no alcohol drinking, and no CCB use, lower WHR, serum TC, LDL-C levels, and PA intensity, and worse sleep quality. However, there was no statistically significant difference in hypertension classification, systolic pressure at ordinary times, diastolic pressure at ordinary times, and course of hypertension between the CI group and the normal cognitive function group in older adults hypertensive patients (*p* < 0.05).

**Table 1:** Baseline differences between older adults hypertensive patients with CI and normal cognitive function.

Variable	CI group (*N* = 518)	Normal cognitive function group (*N* = 1,554)	*p*-value
Basic information			
Age, year	72.58 ± 5.57	72.13 ± 5.92	0.135
Gender, *n* (%)	229 (44.21)	687 (44.21)	1.000
Education level, *n* (%)	364 (70.27)	843 (54.25)	<0.001*
Type of work, *n* (%)	407 (78.57)	936 (60.23)	<0.001*
Smoking, *n* (%)	466 (89.96)	1,390 (89.45)	0.740
Alcohol drinking, *n* (%)	470 (90.73)	1,356 (87.26)	0.034*
Course of hypertension, month	119.00 (40.75, 230.25)	119.00 (48.00, 198.00)	0.907
Classification of hypertension, *n* (%)	44 (8.49)	174 (11.20)	0.193
Systolic pressure at ordinary times, mmHg	140.49 (12.82)	142.24 (12.88)	0.120
Diastolic pressure at ordinary times, mmHg	83.99 (8.34)	83.75 (9.85)	0.348
WHR	0.90 ± 0.06	0.90 ± 0.08	<0.001*
BMI, Kg/m^2^	25.00 ± 3.27	25.23 ± 3.07	0.180
Auxiliary examination			
FBG, mmol/L	6.61 ± 2.40	6.85 ± 2.76	0.095
TG, mmol/L	1.23 (0.86, 1.71)	1.26 (0.93, 1.79)	0.211
TC, mmol/L	4.40 ± 1.17	4.55 ± 1.20	0.013*
HDL-C, mmol/L	1.24 ± 0.40	1.26 ± 0.37	0.082
LDL-C, mmol/L	2.57 ± 0.96	2.76 ± 1.00	<0.001*
Scr, ummol/L	65.45 (54.28, 78.00)	64.60 (55.00, 78.00)	0.891
LVEDD, mm	47.09 ± 6.28	46.59 ± 5.67	0.232
RVEDD, mm	22.04 ± 4.50	22.03 ± 4.04	0.585
LAD, mm	36.66 ± 6.94	36.63 ± 5.95	0.283
Medication use for hypertension			
CCB, *n* (%)	268 (51.74)	680 (43.76)	0.002*
Diuretics, *n* (%)	406 (78.38)	1,248 (80.31)	0.343
ACEI or ARB, *n* (%)	248 (47.88)	715 (46.01)	0.461
Beta-blockers, *n* (%)	394 (76.06)	1,161 (74.71)	0.538
Sympathetic nerve inhibitors, *n* (%)	504 (97.30)	1,526 (98.20)	0.208
Subjective assessment scale			
PA intensity, *n* (%)	138 (26.64)	320 (20.59)	< 0.001*
Sleep quality	78 (15.06)	523 (33.66)	< 0.001*

Data were presented as mean ± SD, median (25th, 75th) or *n* (%). *Statistically significant value (*p* < 0.05). CI, cognitive impairment; WHR, waist-hip ratio; BMI, body mass index; FBG, fasting blood glucose; TG, triglyceride; TC, total cholesterol; HDL-C, high-density lipoprotein cholesterol; LDL-C, low-density lipoprotein cholesterol; Scr, serum creatinine; LVEDD, left ventricular end-diastolic diameter; RVEDD, right ventricular end-diastolic diameter; LAD, left atrium diameter; CCB, calcium channel blocker; ACEI or ARB, angiotensin-converting enzyme inhibitor or angiotensin receptor blocker; PA, physical activity.

Table 2 compares differences in each component of PSQI between the CI group and the normal cognitive function group in older adults hypertensive patients. The results showed that the PSQI total score, subjective sleep quality score, sleep latency score, sleep duration score, sleep efficiency score, sleep disorders score, sleeping pills score, and daytime dysfunction score of the CI group were higher than that of normal cognitive function group in older adults hypertensive patients.

**Table 2:** Differences in each component of PSQI between the CI group and the normal cognitive function group in older adults hypertensive patients.

Variable	CI group (*N* = 518)	Normal cognitive function group (*N* = 1,554)	*P*-value
PSQI total score	8.00 (6.00, 10.00)	7.00 (5.00, 9.00)	<0.001*
Subjective sleep quality score	1.00 (1.00, 2.00)	1.00 (1.00, 1.00)	<0.001*
Sleep latency score	2.00 (1.00, 2.00)	1.00 (1.00, 2.00)	<0.001*
Sleep duration score	1.00 (1.00, 1.00)	1.00 (1.00, 1.00)	<0.001*
Sleep efficiency score	0.00 (0.00, 1.00)	0.00 (0.00, 1.00)	<0.001*
Sleep disorders score	1.00 (1.00, 2.00)	1.00 (1.00, 1.00)	<0.001*
Sleeping pills score	1.00 (0.00, 2.00)	0.00 (0.00, 1.00)	<0.001*
Daytime dysfunction score	2.00 (1.00, 2.00)	1.00 (1.00, 2.00)	<0.001*

Data were presented as median (25th, 75th). *Statistically significant value (*P* < 0.05). PSQI, Pittsburgh Sleep Quality Index; CI, cognitive impairment.

**Figure 1:**
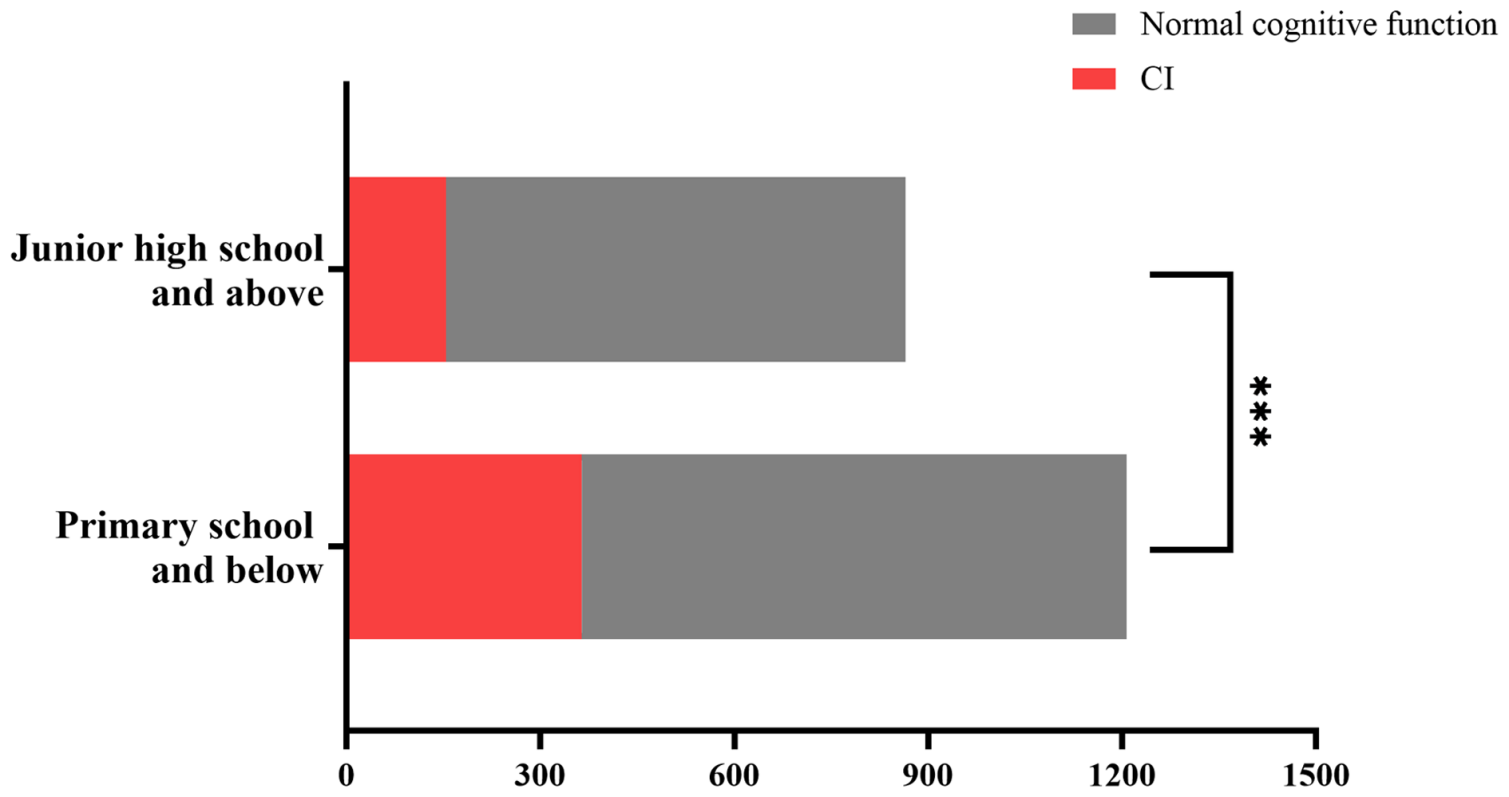
Differences in cognitive function among older adults hypertensive patients with different levels of education. Older adults hypertensive patients with primary school and below had a higher proportion of CI compared with those with junior high school education and above (30.16% vs 17.80%, *P* < 0.001). ***Statistically significant value (*P* < 0.001). CI, cognitive impairment.

Figure 1 analyzes of differences in cognitive function among older adults hypertensive patients with different educational levels using the Chi-square test. The proportion of CI in older adults hypertensive patients with primary school education and below was higher than that in older adults hypertensive patients with junior high school education and above (30.16% vs. 17.80%, *p* < 0.001).

### 3.2 The relationship between sleep quality and cognitive function in older adults hypertensive patients

Table 3 uses logistic regression to analyze the relationship between sleep quality and cognitive function in older adults hypertensive patients. Without adjusting for any factors, sleep quality was positively correlated with CI in older adults hypertensive patients (OR = 2.862, 95%CI: 2.200–3.722, *p* < 0.001), and this correlation still existed in the older adults hypertensive patients with education levels of primary school and below and junior high school and above (OR = 2.633, 95%CI: 1.887–3.673, *p* < 0.001; OR = 2.697, 95%CI: 1.585–4.592, *p* < 0.001). After adjusting for type of work, alcohol drinking, systolic pressure at ordinary times, serum TC and LDL-C levels, LAD, CCB, and PA intensity, sleep quality was also positively correlated with CI in older adults hypertensive patients (OR = 2.565, 95%CI: 1.958–3.360, *p* < 0.001). This relationship still existed in the older adults hypertensive patients with education levels of primary school and below and junior high school and above (OR = 2.468, 95%CI: 1.754–3.473, *p* < 0.001; OR = 2.385, 95%CI: 1.367–4.161, *p* = 0.002).

**Table 3:** The relationship between sleep quality and cognitive function in older adults hypertensive patients.

	CI					
	Total		Primary school and below		Junior high school and above	
	OR (95% CI)	*P*-value	OR (95% CI)	*P*-value	OR (95% CI)	*P*-value
Model 1	2.862 (2.200, 3.722)	<0.001*	2.633 (1.887, 3.673)	<0.001*	2.697 (1.585, 4.592)	<0.001*
Model 2	2.565 (1.958, 3.360)	<0.001*	2.468 (1.754, 3.473)	<0.001*	2.385 (1.367, 4.161)	0.002*

Model 1: crude, no adjustment. Model 2: adjusting for type of work, alcohol drinking, systolic pressure at ordinary times, serum TC and LDL-C levels, LAD, CCB, and PA intensity. *Statistically significant value (*p* < 0.05). CI, cognitive impairment; TC, total cholesterol; LDL-C, low-density lipoprotein cholesterol; LAD, left atrium diameter; CCB, calcium channel blocker; PA, physical activity.

### 3.3 Relationship between each component of PSQI and CI in older adults hypertensive patients

**Figure 2:**
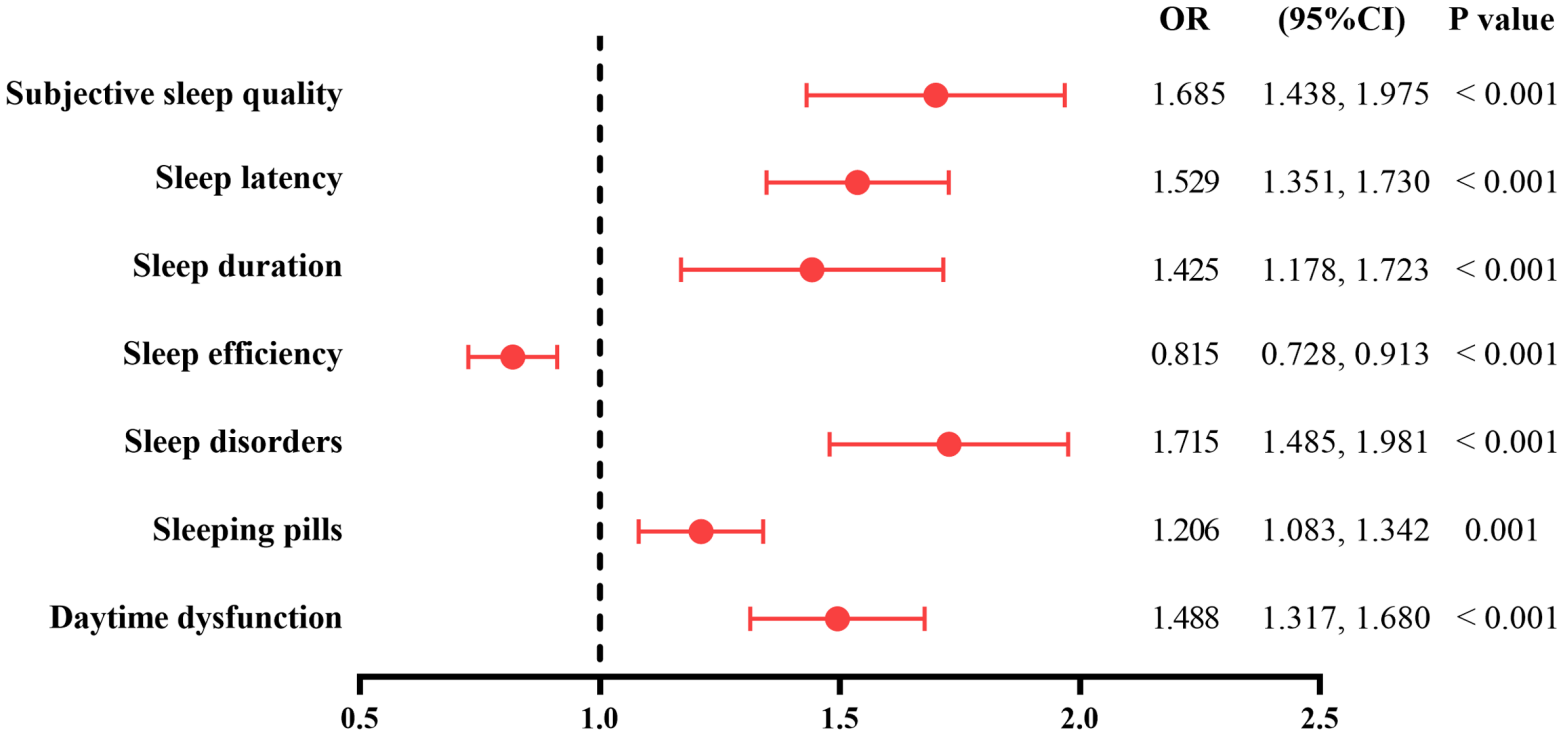
The relationship between each component of PSQI and CI in older adults hypertensive patients. The model was adjusted for education level, type of work, alcohol drinking, systolic pressure at ordinary times, serum TC and LDL-C levels, LAD, CCB, and PA intensity. PSQI, Pittsburgh Sleep Quality Index; CI, cognitive impairment; TC, total cholesterol; LDL-C, low-density lipoprotein cholesterol; LAD, left atrium diameter; CCB, calcium channel blocker; PA, physical activity.

Figure 2 uses multivariate logistic regression analysis to analyze the relationship between each component of PSQI and CI in older adults hypertensive patients. After adjusting for education level, type of work, alcohol drinking, systolic pressure at ordinary times, serum TC and LDL-C levels, LAD, CCB, and PA intensity, the scores of subjective sleep quality (OR = 1.685, 95%CI: 1.438–1.975, *p* < 0.001), sleep latency (OR = 1.529, 95%CI: 1.351–1.730, *p* < 0.001), sleep duration (OR = 1.425, 95%CI: 1.178–1.723, *p* < 0.001), sleep disorders (OR = 1.715, 95%CI: 1.485–1.981, *p* < 0.001), sleeping pills (OR = 1.206, 95%CI: 1.083–1.342, *p* = 0.001), and daytime dysfunction (OR = 1.488, 95%CI: 1.317–1.680, *p* < 0.001) were positively correlated with CI, and the score of sleep efficiency was negatively correlated with CI in older adults hypertensive patients (OR = 0.815, 95%CI: 0.728–0.913, *p* < 0.001).

### 3.4 The correlation between sleep quality and each part of the MMSE score in older adults hypertensive patients

Table 4 shows that the Spearman correlation coefficient is used to study the correlation between sleep quality and each part of the MMSE score in older adults hypertensive patients. The results showed that sleep quality was negatively associated with orientation (r = −0.139, *p* < 0.001), memory (*r* = −0.085, *p* < 0.001), attention and numeracy (*r* = −0.232, *p* < 0.001), recall ability (*r* = −0.131, *p* < 0.001), and language ability (*r* = −0.188, *p* < 0.001) in older adults hypertensive patients.

**Table 4:** Correlation of sleep quality and each part of MMSE scores in older adults hypertensive patients.

	Sleep quality	
	*r* value	*P-*value
Orientation	–0.139	<0.001*
Memory	–0.085	<0.001*
Attention and numeracy	–0.232	<0.001*
Recall ability	–0.131	<0.001*
Language ability	–0.188	<0.001*

*Statistically significant value (*p* < 0.05). PA, physical activity; MMSE, Mini-mental State Examination.

### 3.5 The mediating effect of sleep quality between PA intensity and CI in older adults hypertensive patients

Table 5 uses the Mediation package in R Language to analyze the mediating effect of sleep quality between PA intensity and CI in older adults hypertensive patients. PA intensity was used as the independent variable, sleep quality was the mediator variable, and CI was the outcome variable. In addition, type of work, alcohol drinking, systolic pressure at ordinary times, serum TC and LDL-C levels, LAD, and CCB were included in the model as adjusted variables. The results showed that sleep quality had a partial mediating effect between PA intensity and CI in older adults hypertensive patients (Za * Zb: –17.19339; 95%CI: –0.37312, –0.04194).

**Table 5:** Mediating effect of sleep quality between PA intensity and CI in older adults hypertensive patients.

Variables	Estimate	Standard error	OR (95% CI)	*P-*value	Z_a_ * Z_b_ (95% CI)
PA intensity (X) → sleep quality (M)	–0.20925	0.08320	0.811 (0.689, 0.955)	0.012*	
sleep quality (M) → CI (Y)	0.94197	0.13779	2.565 (1.958, 3.360)	<0.001*	–17.19339 (–0.37312, –0.04194)^#^
PA intensity (X) → CI (Y)	–0.40019	0.09444	0.670 (0.557, 0.806)	<0.001*	

Type of work, alcohol drinking, systolic pressure at ordinary times, serum TC and LDL-C levels, LAD, and CCB as adjusted variables. *Statistically significant value (*p* < 0.05); ^#^Statistically significant value (The 95% CI for the mediation effect does not include 0); X, independent variable; M, mediating variable; Y, dependent variable; Z_a_, estimates of X and M/standard error of X and M; Z_b_, estimates of M and Y/standard error of M and Y; CI, cognitive impairment; PA, physical activity.

## 4 Discussion

The purpose of this study was to investigate the relationship between sleep quality and CI in older adults hypertensive patients. The results of this study showed that poor sleep quality was associated with CI in older adults hypertensive patients, and this correlation still existed in older adults hypertensive patients with education levels of primary school and below and junior high school and above. In each part of the PSQI, the scores of subjective sleep quality, sleep latency, sleep duration, sleep disorders, sleeping pills, and daytime dysfunction were positively correlated with CI, and the score of sleep efficiency was negatively correlated with CI. Sleep quality in older adults hypertensive patients was negatively correlated with orientation, memory, attention and numeracy, recall ability, and language ability in MMSE. In addition, sleep quality partly mediated the association between PA intensity and CI in older adults hypertensive patients.

In this study, the baseline data characteristics of older adults hypertensive patients with CI and those with normal cognitive function were analyzed. The results showed that compared with the normal cognitive function group, the CI group had a higher proportion of no CCB use and work type with physical work mainly. A cross-sectional study found that the total score of MMSE of the Chinese older adults with work type of mental work was significantly higher than that of the Chinese older adults with work type of physical work (36), which might be because work type of mental work could improve the cognitive reserve (CR) of the older adults and thus help them maintain better cognitive function (37). A prospective study found that older hypertensive patients who used CCB had better memory (38). In an epidemiological investigation, alcohol drinking less than 40 g per day in women and less than 80 g per day in men reduced the occurrence of CI (39). This study did not calculate the alcohol consumption of older adults hypertensive patients, but the results of this study showed that older adults hypertensive patients with CI had a higher proportion of no alcohol drinking than those with normal cognitive function. This study also found that the older adults hypertensive patients with CI had lower WHR, serum TC and LDL-C levels, and PA intensity than those with normal cognitive function, which were similar to the results of some studies (28, 40–42).

Previous studies have found that sleep quality affects the cognitive function of patients. Five cross-sectional studies found that poor sleep quality was negatively correlated with MMSE total score in older adults (21); sleep disorders (PSQI total score > 5) were associated with MCI in Chinese community population (43); poor sleep quality was associated with CI in Chinese older adults and centenarians (44, 45); poor sleep quality was associated with CI in older adults hypertensive patients (46); PSQI total score was positively correlated with CI in older adults hypertensive patients (47). In a cross-sectional study using a multistage cluster random sampling method, Insomnia status (Athens Insomnia Scale >6) was associated with a lower total MMSE score in rural older adults (48). A longitudinal survey found that poor sleep quality was associated with CI in Chinese older adults (49). A prospective cohort study found that a higher PSQI total score was associated with CI in older adults men (50). A retrospective cohort study found that poor sleep quality was associated with the development of MCI in Germans (51). Similarly, this study also found that poor sleep quality was associated with CI in older adults hypertensive patients, and this relationship existed in hypertensive patients with education levels of primary school and below and junior high school and above. In each part of the PSQI, the results of this study showed that the scores of subjective sleep quality, sleep latency, sleep duration, sleep disorders, sleeping pills, and daytime dysfunction had a positive correlation with CI, which were similar to the results of previous studies. Two cross-sectional studies have found that poor subjective sleep quality and daytime dysfunction contribute to the occurrence of CI in Chinese older adults (44, 52). A prospective cohort study found that longer sleep latency in Korean older adults with normal cognitive function or MCI was associated with a decline in cognitive function 4 years later (53). Two cross-sectional studies found that sleep duration of 6–7 h, < 6 h, 8–9 h, and ≧ 9 h compared with sleep duration of 7–8 h promoted the occurrence of CI in older adults Chinese (44); the older adults with sleep duration <6 h and > 8 h were more likely to have CI compared with the older adults with 6–8 h of sleep (54). Another prospective study found that older women who slept ≥8 and ≤ 6 h/night had a higher risk for MCI or dementia compared with 7 h/night (55). Both long and short sleep duration contributed to circadian dysfunction and thus contributed to CI (56). This study showed that the score of sleep efficiency had a negative correlation with CI, which was in contrast to the results of previous studies (43, 52). In addition, the results of the present study also found that sleep quality was negatively correlated with orientation, memory, attention and numeracy, recall ability, and language ability in older adults hypertensive patients, which were similar to the results of the following studies. A cross-sectional study using multiple linear regression analysis showed that insomnia (AIS > 6) was negatively correlated with attention and numeracy, memory, and language ability in older adults (48). A community epidemiology study found that PSQI total score was negatively correlated with memory in non-demented older adults (57). In conclusion, poor sleep quality promoted the occurrence of CI and decreased MMSE scores of orientation, memory, attention and numeracy, recall ability, and language ability in older adults hypertensive patients.

The mechanism of the relationship between poor sleep quality and CI is still unclear, and it may be related to the deposition of brain amyloid *β*-protein (Aβ), the decrease of brain-derived neurotrophic factor (BDNF), and cortical atrophy. Aβ caused the decline in cognitive function by causing erroneous neural network activity and impairing synapses between neurons that form and maintain microcircuits that support learning, memory, and other cognitive functions (58). A cross-sectional study found that poor sleep quality was associated with increased brain Aβ in community-dwelling older adults (59). Two cohort studies have shown that poor sleep efficiency and reduced low-frequency < 1 Hz slow waves during non-rapid eye movement sleep promote the accumulation rate of brain Aβ in older adults people with normal cognitive function (60); poor sleep quality in middle-aged and older Koreans increases the deposition of pathological Aβ in the brain (61). BDNF is a neuroprotective factor that promotes long-term memory storage by long-term potentiation of hippocampal glutamatergic neurons and enhances dendritic spine growth and reorganization in response to altered neural activity (62–64). Low levels of BDNF, both in peripheral blood and in cerebrospinal fluid, were associated with cognitive decline (65, 66). A systematic review and meta-analysis found that peripheral BDNF was lower in insomnia patients than in controls (67). A case–control study found that higher insomnia scores were associated with lower serum BDNF levels (68). Animal experimental studies have shown that the mRNA and protein levels of BDNF in the hippocampus of sleep-deprived rats are lower than those of caged control rats (69). Cortical atrophy was related to cognitive function (70), especially posterior cortical atrophy (71). A cohort study found that poor sleep quality was associated with cortical atrophy in community adults (72). A cross-sectional study found that the cortical and subcortical volumes of older adults with poor sleep were smaller than those of older adults with normal sleep (73). Hypertension was a risk factor for CI in the older adults (28), and poor sleep quality was related to the occurrence of CI in older adults patients with hypertension. Therefore, older adults hypertensive patients should maintain good sleep quality.

The results of the present study also showed that the prevalence of CI in older adults hypertensive patients with primary school education and below was significantly higher than that in older adults hypertensive patients with junior high school education and above, which was similar to the results of some studies. Two systematic reviews and meta-analyses found that Chinese hypertensive patients with primary school education and below promoted the occurrence of CI compared with those with junior high school education and above (6); community residents aged 50 and above with education level > 6 years had a lower prevalence of MCI than community residents aged 50 and above with education level ≤ 6 years (74). Two cross-sectional studies have found that Chinese hypertensive patients with education level of junior high school and below have an increased risk of MCI compared with those with education level of junior high school and above (75); Chinese older adults with education levels of junior high school and above and primary school have a lower incidence of CI than illiterate Chinese older adults (76). The mechanism by which higher education level reduces the prevalence of CI in older adults hypertensive patients may be related to better CR. Studies have found that higher levels of education are associated with better CR (77). CR could increase the functional connectivity of the human brain’s large network and promote the formation of better organization of human brain network topology, which improved the ability of the human brain to process information (78), thereby reducing the occurrence of CI (77).

There were few studies on the relationship between PA, sleep quality, and CI in older adults hypertensive patients, but there were some studies in other populations. Two cross-sectional studies have found that sleep quality has a partial mediating effect between physical exercise and CI in older adults with type 2 diabetes (79); sleep quality mediates the relationship between PA and cognitive function in adults aged 50 years or above to a certain extent (80). A randomized controlled study showed that PA in older adults could improve the cognitive ability of older adults by improving sleep quality (81). The present study found that sleep quality mediated part of the mediating effect between PA intensity and CI in older adults hypertensive patients. Therefore, higher PA intensity in older adults hypertensive patients reduced the occurrence of CI in part by improving sleep quality.

Admittedly, this study has several limitations. Firstly, this study was a case–control study and could not determine the causal relationship between sleep quality and CI in older adults hypertensive patients. Therefore, cohort studies are needed to determine this relationship. Second, although a multivariate logistic regression model was used to adjust for confounders in this study, the possibility of residual bias due to other relevant confounders not included cannot be excluded. Third, PSQI is a subjective evaluation scale, which may lead to recall bias due to the older age and CI of the patients. Finally, the cases included in this study were limited to older adults hypertensive patients attending outpatient clinics or wards of hospitals in Shandong Province, China, and future studies with large samples and multiple regions are needed. However, this study found that poor sleep quality can promote the occurrence of CI in older adults hypertensive patients, and this relationship still existed in older adults hypertensive patients with education levels of primary school and below and junior high school and above. In addition, sleep quality had a partial mediating effect between PA intensity and CI in older adults hypertensive patients. Therefore, it is suggested that older adults hypertensive patients should maintain good sleep quality.

## 5 Conclusion

Poor sleep quality promoted the occurrence of CI in older adults hypertensive patients, and this relationship also existed in older adults hypertensive patients with education levels of primary school and below and junior high school and above. The mechanism may be related to the deposition of Aβ in the brain, decreased BDNF, and cortical atrophy. In addition, sleep quality partly mediated the mediating effect between PA intensity and CI in older adults hypertensive patients. Therefore, this study suggests that older adults hypertensive patients should improve sleep quality by improving subjective sleep quality, shortening sleep latency, maintaining sleep duration for about 7 h, and so on to reduce the occurrence of CI.

## Data Availability

The raw data supporting the conclusions of this article will be made available by the authors, without undue reservation.
